# Primary aldosteronism complicated by early-onset heart failure in a young male with a coexisting *DMD* variant: A case report and literature review

**DOI:** 10.1097/MD.0000000000045443

**Published:** 2025-11-07

**Authors:** Xiaoxiao Song, Siwei Qian, Minyue Jia, Hanxiao Yu, Minzhi He, Hao Yu, Ka I. Cheok, Liya Lin, Zhichao Dong, Xin Pan, Chao Zheng, Jiaming Wen, Xiaohong Xu

**Affiliations:** a Department of Endocrinology, The Second Affiliated Hospital, Zhejiang University School of Medicine, Hangzhou, Zhejiang Province, China; b Zhejiang University School of Medicine, Hangzhou, Zhejiang Province, China; c Department of Ultrasonography, The Second Affiliated Hospital, Zhejiang University School of Medicine, Hangzhou, Zhejiang Province, China; d Clinical Research Center, The Second Affiliated Hospital, Zhejiang University School of Medicine, Hangzhou, Zhejiang Province, China; e Department of Vascular Surgery, The Second Affiliated Hospital, Zhejiang University School of Medicine, Hangzhou, Zhejiang Province, China; f Department of Medical Genetics, The Second Affiliated Hospital, Zhejiang University School of Medicine, Hangzhou, Zhejiang Province, China; g Department of Urology, The Second Affiliated Hospital, School of Medicine, Zhejiang University, Hangzhou, Zhejiang Province, China; h Department of Endocrinology, The First People’s Hospital of Xiaoshan District, Hangzhou, Zhejiang Province, China.

**Keywords:** adrenalectomy, case report, *DMD* gene, heart failure, primary aldosteronism

## Abstract

**Rationale:**

Heart failure (HF) is slightly more common in primary aldosteronism (PA) than in essential hypertension, but early-onset HF remains rare. In such cases, underlying genetic cardiomyopathies should be considered. Autonomously secreted aldosterone and activation of the renin-angiotensin-aldosterone system can lead to extremely high aldosterone levels, worsening cardiac function and creating major therapeutic challenges.

**Patient concerns:**

A 39-year-old male presented with progressive chest tightness and shortness of breath for 4 months. He had a 7-year history of hypertension and persistent hypokalemia. Electrocardiogram revealed a markedly reduced left ventricular ejection fraction of 18.3%.

**Diagnosis and interventions:**

The patient was diagnosed with PA based on elevated plasma aldosterone concentration, an increased aldosterone-to-renin ratio, and a positive captopril challenge test. Computed tomography and adrenal vein sampling indicated unilateral PA. After initial HF management, the patient underwent laparoscopic adrenalectomy for PA treatment.

**Outcomes:**

According to the primary aldosteronism surgical outcome consensus criteria for postoperative evaluation of PA, complete biochemical remission (normalization of aldosterone-to-renin ratio and potassium) and partial clinical remission (stable blood pressure with reduced antihypertensive medication) were achieved 1 month postoperatively and have been maintained since. At the 8-month follow-up, his left ventricular ejection fraction had improved to 45.4% and BNP levels normalized. Whole-exon sequencing revealed a missense mutation of the dystrophin (*DMD*) gene. Certain *DMD* mutations are linked to X-linked dilated cardiomyopathy with absent or subclinical skeletal muscle involvement. Sanger sequencing confirmed the hemizygous mutation in the proband. The final diagnosis was poorly controlled PA with early-onset HF, potentially influenced by a coexisting *DMD* gene missense mutation that may modify both the onset and severity of PA-related HF.

**Lessons:**

Early recognition and surgical treatment of PA with early-onset HF can substantially improve cardiac function, even in the presence of genetic susceptibility to cardiomyopathy. This case underscores the need to consider underlying cardiac genetic disorders in PA patients with atypical or early-onset HF and raises the hypothesis that the identified *DMD* variant may serve as a potential genetic modifier of HF severity in the context of PA.

## 1. Introduction

Aldosteronism primarily presents as hypertension caused by sodium retention, potassium excretion, and increased blood volume, with or without hypokalemia. Elevated plasma aldosterone levels can be classified as primary or secondary. Primary aldosteronism (PA), which accounts for 5% to 10% of patients with hypertension,^[1,2]^ is characterized by autonomous adrenal secretion of aldosterone with suppressed renin activity, and represents a leading cause of secondary hypertension. In contrast, secondary aldosteronism refers to pathologically elevated aldosterone levels resulting from activation of the renin-angiotensin-aldosterone system (RAAS).

Despite the relatively high prevalence of PA, its diagnosis is often delayed, which can lead to markedly cardiovascular complications, including heart failure (HF). Notably, congestive HF driven by PA can reduce effective blood volume, thereby provoking the RAAS and further triggering secondary aldosteronism further.^[3–5]^ However, only a few cases of PA complicated by early-onset HF with reduced ejection fraction have been reported, and evidence regarding the optimal treatment strategy and its effectiveness in managing PA with this HF remains limited.^[6,7]^ Herein, we report a young patient with PA complicated by HF in the context of an underlying primary cardiac genetic disorder, underscoring the interaction between genetic susceptibility and the endocrine environment. The coexistence of PA and secondary aldosteronism resulted in extremely high aldosterone levels, far exceeding those typically observed in PA alone. The direct toxic effects of such elevated aldosterone levels on vital organs, combined with cardiac insufficiency, posed considerable therapeutic and surgical challenges. Following multidisciplinary evaluation and treatment, the young patient achieved complete biochemical remission and partial clinical remission of PA after laparoscopic adrenalectomy, with significant improvement in cardiac function. The ethics statement for this case has been properly addressed in the declarations section at the end of the text.

## 2. Case report

### 2.1. Patient information

A 39-year-old man was admitted with a 4-month history of chest distress, dyspnea, and abdominal distension. His symptoms worsened with minimal exertion and prevented him from lying flat at night. He gradually developed bilateral lower limb edema but denied chest pain, dizziness, or fever. He had not received regular treatment. He had a 7-year history of hypertension and hypokalemia, without coronary artery disease. His blood pressure (BP) peaked at 185/110 mm Hg and remained 150 to 160/100 mm Hg on amlodipine 10 mg daily. Serum potassium (reference range: 3.5–5.3 mmol/L) dropped to 1.2 mmol/L and fluctuated around 3.0 mmol/L despite potassium chloride 0.75 g 3 times daily. The patient did not seek further evaluation for the cause of hypertension. He also reported poor appetite and a 5-kg weight loss. Family history revealed hypertension without hypokalemia in his father, while his mother had no history of either condition.

On admission, physical examination revealed a respiratory rate of 18 breaths/min, a pulse rate of 95 beats/min, an average 24-hour BP of 157/114 mm Hg, and a body mass index of 22.9 kg/m². Cardiac examination demonstrated enlarged heart with a regular rhythm and no audible murmurs. Chest auscultation detected fine moist crackles at the lung bases. Pitting edema was observed in both lower extremities. Notably, physical features such as moon facies, buffalo hump, and abdominal striae were absent. Electrocardiogram (ECG) indicated left atrial enlargement and a first-degree atrioventricular block, while chest radiography revealed cardiomegaly characterized by an enlarged cardiac silhouette.

Post-admission laboratory tests (Table S1, Supplemental Digital Content, https://links.lww.com/MD/Q458) revealed elevated levels of pro-BNP (7297 pg/mL, reference range: <125 pg/mL), stage 3 renal insufficiency (creatinine: 187.3 µmol/L; estimated glomerular filtration rate [eGFR]: 38.08 mL/min). The LDL level was 1.9 mmol/L (reference range:<3.4mmol/L). Echocardiography indicated global cardiac enlargement, biventricular systolic dysfunction, and a left ventricular ejection fraction (LVEF) of 18.3% (reference range: 52.6–76.2%). Ultrasonography revealed spongy nephrotic changes in both kidneys and hypoechoic nodules in the right adrenal gland. Based on the above clinical manifestations and examinations, the initial diagnosis included HF with New York Heart Association class IV, stage 3 chronic kidney disease with bilateral medullary sponge kidney, grade 3 hypertension, and hypokalemia. With oxygen support and potassium chloride supplementation at 3.0 g/d, the patient’s antihypertensive and anti-HF regimen was adjusted to include amlodipine 5 mg/d, spironolactone 60 mg/d, and an angiotensin receptor-neprilysin inhibitor (ARNI), sacubitril/valsartan, which was titrated from 100 mg/d to 200 mg/d. As a result, the patient’s BP (140/90 mm Hg), pro-BNP (1261 pg/mL), creatinine (135 µmol/L), and potassium (3.8 mmol/L) were brought under control.

### 2.2. Diagnostic assessment

**Figure 1.**
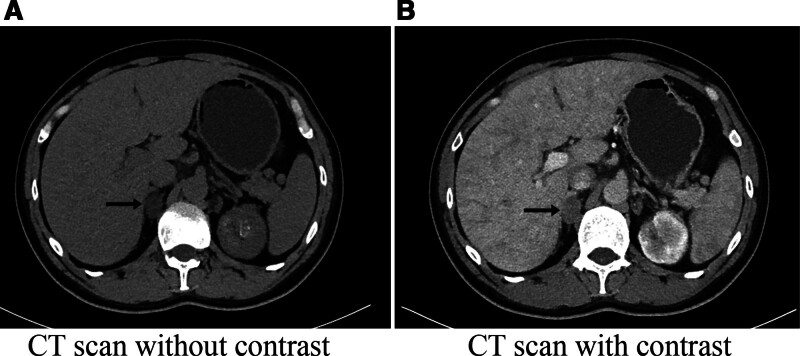
Abdominal computerized tomography (CT) image. The abdominal CT scan reveals a 23 mm × 16 mm nodule in the right adrenal gland. (A) The nodule appears as a relatively low-density area (arrows). (B) Mild enhancement is observed following contrast administration (arrows).

Considering the patient’s long history of hypertension and laboratory results indicative of hypokalemia and renal potassium loss, PA was suspected at admission. Accordingly, endocrine testing was performed without delay, which revealed the following results: the plasma aldosterone concentration (PAC) was 6420.0 pg/mL (reference range: 30.0–353.0 pg/mL), the direct renin concentration (DRC) was 14.9 µIU/mL (reference range: 4.4–46.1 µIU/mL), and the plasma aldosterone-to-renin ratio (ARR) was 430.87 pg/µIU (reference range <37). Additionally, blood cortisol levels were measured at different times of the day: 722.0 nmol/L at 8 am (reference range: 166.0–507.0 nmol/L) and 663.8 nmol/L at 4 pm (reference range: 73.8–291.0 nmol/L). Meanwhile, ACTH levels were 36.5 pg/mL at 8 am (reference range: 7.2–63.3 pg/mL) and 49.9 pg/mL at 4 pm These results from the hypothalamic-pituitary-adrenal axis were considered secondary to stress. Catecholamines and their metabolites were found to be within normal limits. To further confirm the diagnosis, a captopril challenge test was performed, and the positive result supported the diagnosis of PA: the 60- and 120-minute tests after the captopril challenge showed that the PAC and ARR remained elevated (3010 pg/mL [baseline], 2470 pg/mL [1 hour] and 2370 pg/mL [2 hours] respectively). The computerized tomography scanning of the adrenal glands revealed a 23 mm  × 16 mm nodule in the medial branch of the right adrenal gland. This nodule showed mild enhancement after contrast administration and exhibited characteristics consistent with an adenoma. In contrast, the left adrenal gland appeared normal (Fig. 1). To determine whether there was lateralized secretion of aldosterone, bilateral adrenal vein sampling was performed. The results indicated that the right adrenal gland was the primary source of aldosterone secretion (lateralization index, LI: 47.3) for PA, with concomitant suppression of aldosterone secretion from the left adrenal gland. The treatment regimen for acute HF and PA was maintained as before, except that the spironolactone dose was titrated to 40 mg twice daily. After 4 weeks of treatment, the patient’s cardiac function improved, and dobutamine stress ECG revealed that the patient’s heart retained a certain degree of regional contractile reserve, sufficient to tolerate surgical stress.

### 2.3. Therapeutic intervention and outcomes

**Figure 2.**
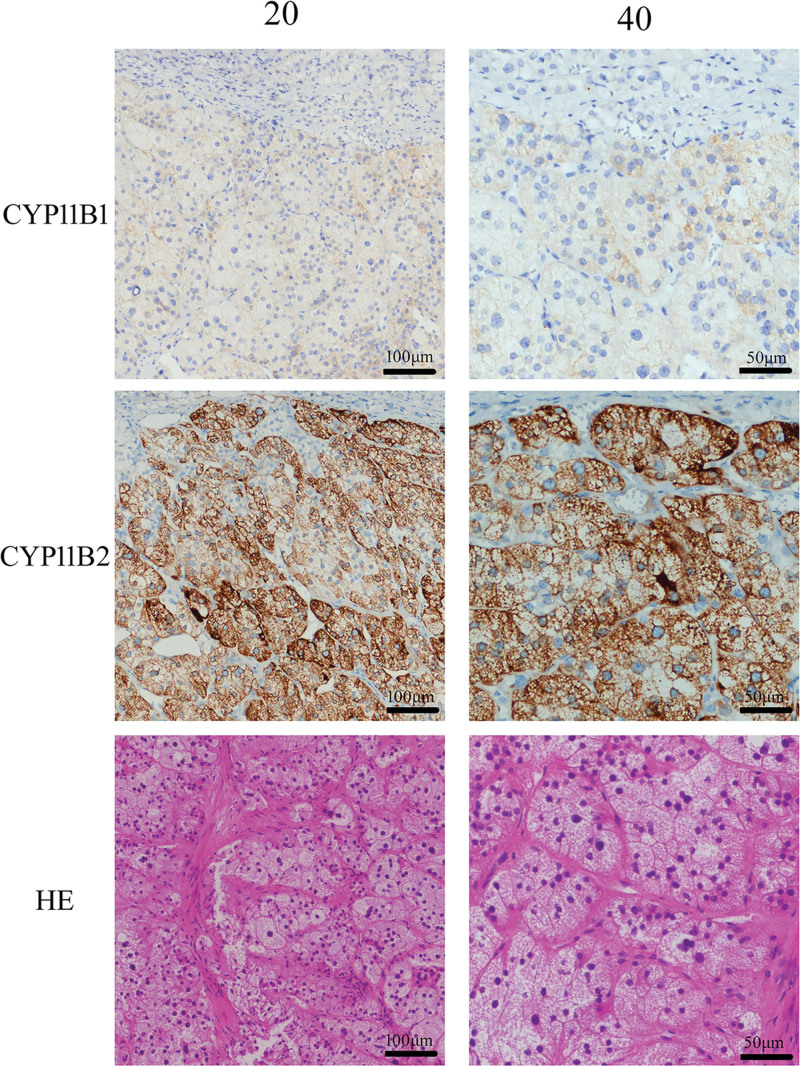
Immunohistochemical analysis of the adrenal adenoma. Hematoxylin and eosin staining, as well as immunohistochemistry for CYP11B1 and CYP11B2, were performed on the patient’s adrenal adenoma tissue.

Subsequently, following a multidisciplinary team discussion, the patient was referred to the urology department for a laparoscopic right adrenalectomy to reduce the aldosterone load. The postoperative pathology revealed a right adrenocortical adenoma. Immunohistochemical results showed: Melan-A+, CYP11B1 partially weak+, CYP11B2+, and Ki-67 1%+ (Fig. 2). This finding definitively confirmed the presence of a right-sided aldosterone-producing adenoma (APA). One month post-surgery, complete biochemical remission (ARR: 3.2, PAC: 219.0 pg/mL, DRC: 68.5 µIU/mL; potassium: 4.6 mmol/L) and partial clinical remission (BP: 120/80 mm Hg) were achieved according to international primary aldosteronism surgical outcomes standards.^[8]^ Renal function also showed improvement (creatinine 139.0 μmol/L; eGFR 43 mL/min; urinary albumin-to-creatinine ratio 46.9 mg/g, reference range: <25.0 mg/g). While pro-BNP was 370 pg/mL (reference range: <125 pg/mL), troponin-T level was within the normal range. The ECG revealed normal and the LVEF value had recovered to 39.3% (reference value: 52.6–76.2%). After surgery, the antihypertensive regimen was reduced to ARNI (sacubitril/valsartan) 100 mg/d, metoprolol 23.75 mg/d, and furosemide 10 mg/d. At the 8-month follow-up, complete biochemical remission and partial clinical remission were maintained according to the primary aldosteronism surgical outcomes standards. The ARR remained at 3.66 (PAC: 359.0 pg/mL; DRC: 98.2 µIU/mL), potassium level was 4.4 mmol/L, and BP was maintained within the normal range (around 125/85 mm Hg). Renal function showed that creatinine was 182.9 µmol/L and eGFR was 41 mL/min. HbA1c was 5.4%, and BNP was within the normal range at 5.5 pg/mL. At 8 months post-surgery, cardiac ultrasound demonstrated that the LVEF had improved to 45.4%. The left ventricular internal diameter at end-diastole was 54.1 mm (reference range: 38.4–54.0 mm), and the left ventricular end-diastolic volume was 95.8 mL (reference range: 45.9–127.5 mL), both values approaching the normal range. Longitudinal variations in LVEF, PAC, ARR, serum potassium, pro-BNP and BP at baseline, postoperative, and during follow-up are summarized in Table 1. Detailed echocardiographic data and images are shown in Table 2 and Figure S1, Supplemental Digital Content, https://links.lww.com/MD/Q459. Other cardiac indices also improved, for example the left ventricular global longitudinal strain, which increased from −9.00% preoperatively to −13.71% at 4 months postoperatively. Unlike conventional LVEF, left ventricular global longitudinal strain, derived from speckle tracking echocardiography, provides an earlier and more sensitive marker of cardiac dysfunction, allowing the detection of both reversible subclinical systolic impairment and overt cardiac involvement in PA.^[3,9]^ The final regimen for this patient was updated to ARNI (sacubitril/valsartan) at 25 mg twice daily, and beta-adrenergic blocker (metoprolol) at 47.5 mg once daily, and the mineralocorticoid receptor antagonist (MRA; finerenone 20 mg) once daily. The patient is satisfied with the improvement in heart function.

**Table 1:** Longitudinal changes in left LVEF, PAC, ARR, serum potassium, and blood pressure at baseline, post-surgery, and follow-up.

Parameter	At presentation	Pre-adrenalectomy	1 mo postoperative	4 mo postoperative	8 mo postoperative
Blood pressure, mm Hg	160/121	140/90	120/80	120/75	125/85
Serum potassium, mmol/L	2.6 ↓	3.34↓	4.6	4.51	4.4
PAC, pg/mL	6420↑	3590↑	219	128	359↑
DRC, µIU/mL	14.9	55.6↑	68.5↑	57.6↑	98.2↑
ARR, pg/µIU	430.87↑	64.57↑	3.2	2.22	3.66
NT-proBNP, pg/mL	7297 ↑	1261↑	370↑	184↑	110
BNP, pg/mL	–	–	–	9.8	5.5
LVEF, %	18.3↓	37.8↓	39.3↓	48.5↓	45.4↓

ARR = aldosterone-to-renin ratio, BNP = B-type natriuretic peptide, DRC = direct renin concentration, LVEF = left ventricular ejection fraction, NT-proBNP = N-terminal pro-B-type natriuretic peptide, PAC = plasma aldosterone concentration.

**Table 2:** Serial measurements on echocardiography, before and after adrenalectomy.

Parameter	Before adrenalectomy		After adrenalectomy			Normal value
	March 2023	April 2023	May 2023	August 2023	December 2023	
LVEF (Simpson’s), %	18.3	37.8	39.3	48.5	45.4	52.6–76.2
IVSd, mm	12.4	11.1	11.7	12.6	13.1	6.4–11.4
LVIDd, mm	62	63.5	61.7	56.1	54.1	38.4–54
LVPWd, mm	12.5	11.2	11.7	12.2	11.4	6.3–11.1
IVSs, mm	13.5	14.4	14.8	14.9	–	9–16
LVIDs, mm	56.9	52.7	50.4	45.4	36.1	22.6–38.6
LVPWs, mm	15.7	14.6	16.1	13.7	–	8.8–16.2
LVEDV, mL	186	160	152	152	95.8	45.9–127.5
LVMI, g/m^2^	192.4	172.9	175.6	162.5	143.6	50–102

Normal value from reference: echocardiographic measurements in normal Chinese adults (EMINCA).

IVSd = interventricular septal thickness at diastole, IVSs = interventricular septum end-systole, LVEDV = left ventricular end-diastolic volume, LVEF = left ventricular ejection fraction, LVIDd = left ventricular internal diameter end-diastole, LVIDs = left ventricular internal diameter end-systole, LVPWd = left ventricular posterior wall dimensions, LVPWs = left ventricular posterior wall end-systole.

### 2.4. Genetic analysis

**Figure 3.**
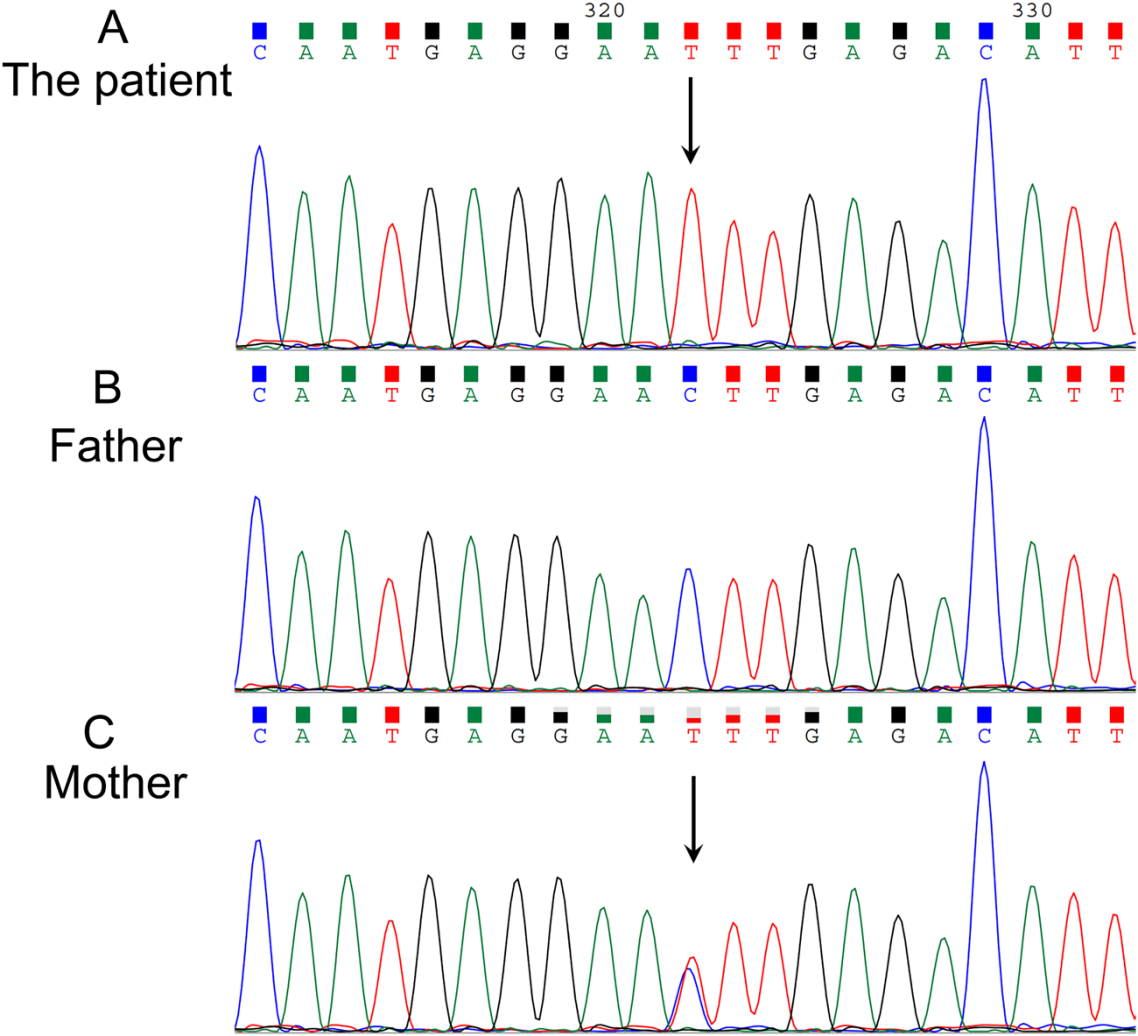
Chromatograms displaying the results of NGS and confirmation by Sanger sequencing. (A) The nucleotide change c.4030C>T in exon 29 of the *DMD* gene, resulting in an amino acid substitution from leucine to phenylalanine at position 1344 in the protein, identified in this patient. (B) The wild type *DMD* exon 29 sequence observed in the patient’s father. (C) The same mutant nucleotide change (c.4030 C>T) and amino acid substitution identified in the patient’s mother. DMD = Duchenne muscular dystrophy.

As this patient lacked significant predisposing risk factors for HF and had no history of coronary atherosclerotic heart disease, whole-exome sequencing was performed to explore the causes of early-onset HF. A hemizygous missense mutation was identified in the dystrophin (*DMD*) gene, exon 29, specifically at nucleotide position 4030 (NM_004006.3; c.4030C>T), resulting in the substitution of leucine with phenylalanine at protein position 1344 (p. Leu1344Phe), which is predicted to impact the protein function. Sanger sequencing confirmed the hemizygous mutation in the proband, heterozygosity in his mother, and normal status in his father (Fig. 3). *DMD* gene variants, which disrupt the function of dystrophin protein, cause a spectrum of X-linked dystrophinopathies. Duchenne muscular dystrophy (DMD) is the most severe form, presenting in early childhood with rapidly progressive muscle weakness and cardiomyopathy. Becker muscular dystrophy (BMD) is a milder allelic disorder with later onset and slower progression. In contrast, X-linked dilated cardiomyopathy (XLDCM) manifests primarily as a primary cardiomyopathy in adolescent or young adult males, with minimal or absent skeletal muscle disease. Clinically, the proband presented with early-onset HF but no skeletal muscle weakness, whereas his mother was asymptomatic with a normal echocardiographic EF, consistent with the X-linked recessive inheritance pattern. Given the absence of skeletal muscle involvement and the predominance of cardiac manifestations, the phenotype in this patient is most consistent with XLDCM.^[10,11]^ Although the p.Leu1344Phe mutation was not located within the core structural region of dystrophin, it may destabilize the protein and act as a modifier. Thus the final diagnosis was poorly controlled PA with early-onset HF, potentially influenced by a coexisting *DMD* gene missense mutation that may modify both the onset and severity of PA-related HF.^[11]^

## 3. Discussion and conclusions

**Figure 4.**
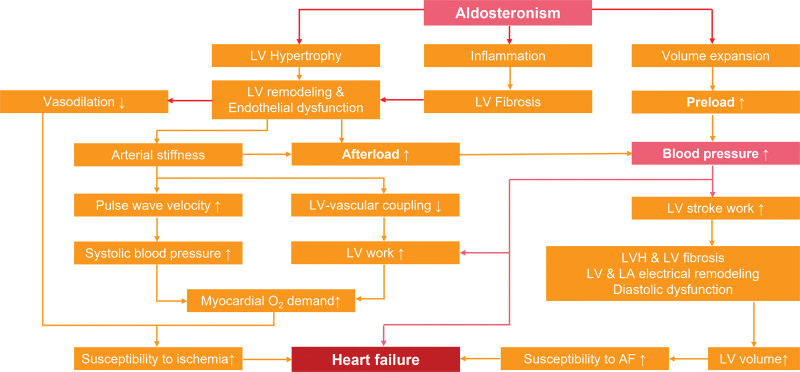
Pathophysiological mechanism of heart failure caused by primary aldosteronism. AF = atrial fibrillation, LA = left atrium, LV = left ventricle, LVH = left ventricle with hypertrophy.

PA is defined by autonomous aldosterone overproduction from the adrenal cortex. Beyond its classical effects of hypertension and hypokalemia, excess aldosterone drives maladaptive cascades via pro-oxidative, pro-inflammatory, and pro-fibrotic mechanisms, leading to target-organ damage.^[12]^ Notably, in patients with PA excess aldosterone significantly increase the risk of cardiotoxic effects, including left ventricular hypertrophy, coronary artery disease, HF, and arrhythmia.^[13–15]^ The mechanisms of PA-induced cardiac damage, particularly HF, are summarized in Figure 4 and involve several key points: First, diverse cell types within the heart, including fibroblasts and cardiomyocytes, express the mineralocorticoid receptor (MR).^[16]^ Elevated levels of aldosterone can activate the MR, leading to ventricular remodeling characterized by hypertrophy and myocardial fibrosis. These changes, in conjunction with endothelial dysfunction and volume expansion, cause elevated BP, impaired vasodilation, increased left ventricle workload, accelerated atherosclerosis and greater myocardial oxygen demand, ultimately predisposing to cardiac fibrillation and HF.^[17–20]^ Beyond MR-dependent mechanisms, recent studies have demonstrated that excessive aldosterone can also cause cardiovascular damage through MR-independent, non-genomic effects, such as activation of phospholipase C, cyclic adenosine monophosphate production, and nitric oxide production.^[21,22]^ For instance, Bunda et al identified a novel MR-independent signaling pathway mediated by Galpha13 that promotes aldosterone-induced elastogenesis in cardiac fibroblasts.^[23]^

In patients with PA and concomitant HF, the diagnosis of PA is often overlooked because of elevated renin levels. Impaired hepatic clearance of aldosterone and activation of the RAAS secondary to reduced blood volume further elevate the already high PAC. Paradoxically, renin levels, which are typically suppressed in PA, also rise as a result of RAAS activation. Although DRC is elevated, the degree of hyperaldosteronism in these patients is more pronounced, and the ARR therefore remains a valuable screening tool for diagnosing PA in the setting of HF. Clinicians should thus be cautious not to exclude PA solely on the basis of elevated DRC levels. For confirmatory diagnosis, the captopril test is generally recommended, whereas the saline infusion test is contraindicated in PA patients with HF. Previously reported cases of concomitant PA and HF are summarized in Table 3 ^[24–28]^ and Table 4,^[6,7,25,29–33]^ which list the relevant screening indicators in these patients.

**Table 3:** Cases of PA combined with HF (drug treatment for PA).

Author (year)		Sarguroh, T.^[24]^	Chen, Z.^[25]^	Meyhöfer, S.^[26]^	Zoltowska, D. M.^[27]^	Sugishita, K.^[28]^
Age (yr), gender		49, male	33, male	60, female	47, female	48, male
Temporal relationship between PA and HF diagnosis		Concurrently	Concurrently	Concurrently	Concurrently	Concurrently
First symptoms		Shortness of breath, bilateral lower extremity edema	Nocturnal dyspnea, exertional dyspnea	Shortness of breath	Chest pain	Leg edema, abdominal distension
BP (mm Hg)		232/177	106/64	160/100	Hypertension	176/122
Serum potassium (mmol/L)		3.3	4.8	–	3.1	2.8
PAC		27.3 ng/dL	251.3 pg/mL	558 ng/L	17 ng/dL	447 pg/mL
PRA (ng/mL/h)		0.7	0.04	/	0.3	0.4
ARR (n < 20)		39	627.5	1577	>20	1117.5
Size of adrenal nodule (CT)		No abnormal findings	30*22 mm (left)	–	Mild bilateral nodular enlargement	No abnormal findings
AVS		–	No dominant secretion	–	–	–
Therapy		Spironolactone, sacubitril/ valsartan, carvedilol, torsemide	Spironolactone, metoprolol, trimetazidine, telmisartan	Spironolactone, doxazosin	Enalapril, aspirin, metoprolol succinate, atorvastatin, spironolactone	Spironolactone, bisoprolol, telmisartan, furosemide, and warfarin
LVEF (%)	Pre-therapy	10–15	30	58	40–45	45
	Post-therapy	–	45	–	normal	49
Follow-up time (mo)		–	11	3	4	12

ARR = aldosterone-to-renin ratio, AVS = adrenal vein sampling, BP = blood pressure, CT = computed tomography, HF = heart failure, LVEF = left ventricular ejection fraction, PA = primary aldosteronism, PAC = plasma aldosterone concentration.

**Table 4:** Cases of PA combined with HF (surgical treatment for PA).

Author (year)		Hoshi, S.^[29]^	Chen, Z.^[25]^	Imamura, Y.^[30]^	Zhang, J.^[31]^	Sato, S.^[6]^	Alvarez, C.^[7]^	Pemayun, T. G. D.^[32]^	Savoriti, C.^[33]^
Age (yr), gender		61, male	43, male	45, male	44, male	57, male	53, male	33, female	54, female
Temporal relationship between PA and HF diagnosis		Concurrently	Concurrently	Concurrently	Concurrently	HF occurred 5 yr before PA	Concurrently	HF occurred after PA	HF occurred 1 yr before PA
BP (mm Hg)		Hypertension	142/110	140/70	172/93	137/102	163/103	170/115	210/105
Serum potassium (mmol/L)		2.5	4.5	3.6	2.05	2.7	2.6	2.2	1.8
PAC		661 pg/mL	185.5 pg/mL	–	18.7 ng/dL	1804 pg/mL	154 ng/dL	56 ng/mL	16.17 ng/dL
PRA (ng/mL/h)		< 0.1	0.46	–	0.06	0.2	<2.1	0.13	0.08
ARR (n < 20)		6610	403.2	–	>20	9002	73.3	430.7	202.17
Size of adrenal nodule (CT)		25 mm (left)	32 mm (left)	30 mm (right)	20 mm (left)	42 mm (right)	24 mm (left)	40 mm (right)	20 mm (left)
AVS		Bilateral PA	–	Right dominant secretion	–	Right dominant secretion	Left dominant secretion	–	–
Pathology		APA	APA	APA	APA	APA	–	APA	APA
LVEF (%)	Pre-therapy	–	30	26	39	20	10–15	17	30
	Post-therapy	–	51	42	52	67	–	56	50
Follow-up time (mo)		24	12	3	2	24	–	18	6

ARR = aldosterone-to-renin ratio, AVS = adrenal vein sampling, BP = blood pressure, CT = computed tomography, HF = heart failure, LVEF = left ventricular ejection fraction, PA = primary aldosteronism, PAC = plasma aldosterone concentration.

On the other hand, in patients with PA and HF, treatment with calcium channel blockers (CCBs), MRAs, and angiotensin receptor blockers (ARBs) generally increases renin and lowers the ARR, thereby predisposing to false negative rather than false-positive results. In this case, drug withdrawal was not feasible due to acute HF; nevertheless, the ARR remained elevated. According to the latest ENDO guidelines, persistence of an elevated ARR despite these medications provides strong evidence of autonomous aldosterone secretion, as such agents would ordinarily suppress the ratio. Moreover, previous studies have demonstrated that confirmatory testing performed under angiotensin-converting enzyme inhibitors, ARBs, CCBs, or β-blockers remains valid when aldosterone secretion is autonomous. For example, a saline infusion test yielding a post-infusion PAC ≥ 240 pmol/L (87pg/mL) has been shown to provide concordant results with testing after drug withdrawal. In our patient, despite concomitant therapy with an ARB, a CCB, and an MRA, the post-captopril PAC remained >2000 pg/mL, far exceeding diagnostic thresholds. Although renin was not fully suppressed due to coexisting HF and the use of interfering medications, such a markedly elevated PAC relative to renin is consistent with autonomous aldosterone secretion and fulfills guideline-based diagnostic criteria. Therefore, the absence of drug withdrawal does not compromise diagnostic validity in this setting.^[1,34]^ Subsequently, a computerized tomography-enhanced scan of the adrenal gland and bilateral adrenal vein sampling confirmed the presence of PA subtype of a unilateral PA.

Given the patient’s young age and long-life expectancy, the multidisciplinary team recommended surgical intervention to potentially reverse cardiac dysfunction. At postoperative follow-up, marked recovery of cardiac function was observed, with normalization of the ECG, BNP levels, and improvement in LVEF. This improvement was likely attributable to reductions in aldosterone levels and BP, together with reversal of left ventricular remodeling and attenuation myocardial fibrosis.^[35,36]^ Considering the cardiotoxic effects of sustained aldosterone excess and hypertension,^[14,37]^ patients with PA complicated by secondary aldosteronism in the setting of HF exhibit higher aldosterone levels than those with PA alone, thereby conferring greater cardiovascular risk and comorbidities.^[38,39]^ Accordingly, in cases of PA complicated by HF, we recommend early management and surgical intervention, which directly lowers aldosterone levels, addresses the underlying cause, and promotes recovery of cardiac function.

Compared with essential hypertension, Huang et al found that patients with APA who underwent surgical treatment had significantly lower rates of HF and mortality.^[40]^ In contrast, Hundemer et al reported that MRAs were associated with higher cardiometabolic risk and mortality compared with patients with essential hypertension or surgically cured PA.^[41]^ When comparing the 2 treatment approaches for PA, surgical intervention appears to provide significant benefits in improving HF outcome relative to MRA therapy. For example, Nezu et al demonstrated enhanced cardiac function following laparoscopic adrenalectomy.^[42–44]^ Overall, when PA is complicated by HF, we recommend surgical treatment to reduce aldosterone burden rather than pharmacological treatment with MRAs, particularly in patients with APA and even in those with idiopathic adrenal hyperplasia at high risk of cardiovascular events.^[45]^

Moreover, the toxic effects of aldosterone extend beyond the cardiovascular system to other organs. This increases the risk of stroke, renal failure, obstructive sleep apnea syndrome, and disorders of fat metabolism.^[13]^ Therefore, surgical intervention can also achieve long-term target organ protection for patients with PA. For instance, Katabami et al reported that surgical treatment improved the eGFR compared to pharmacological treatment.^[46]^ Similarly, Chang et al found that surgical treatment was associated with a lower risk of stroke compared to patients with essential hypertension.^[47]^

In summary, despite the inherent surgical risks in HF patients, early surgical intervention is still considered a reasonable approach given its therapeutic benefits. Previously reported cases of concurrent PA and HF are summarized in Tables 3 and 4, which list the treatment plans, and improvements in cardiac function.

**Figure 5.**
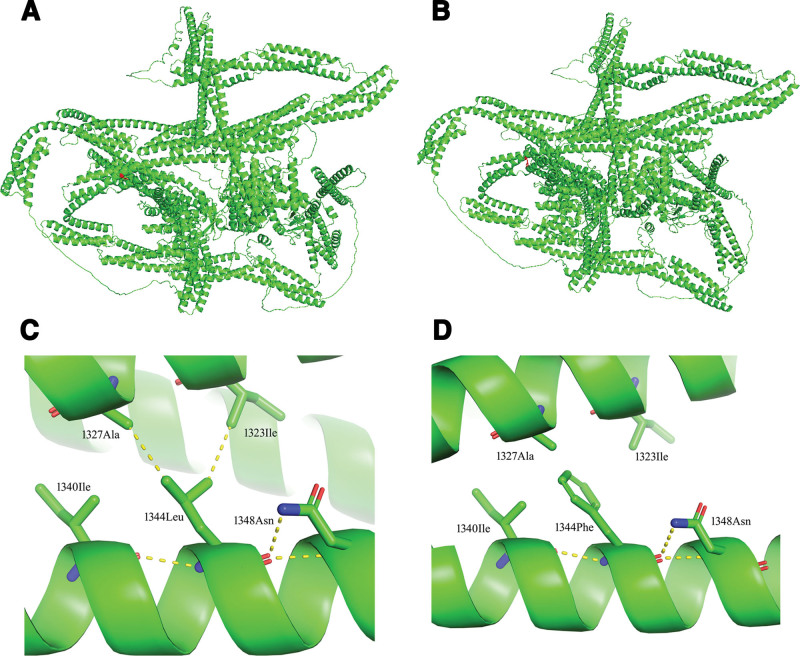
Three-dimensional model of dystrophin proteins and close-up steric view of the p.Leu1344Phe substitution. (A) and (B) The structure of human dystrophin proteins before and after the p.Leu1344Phe substitution, with the red sphere indicating the location of the substituted amino acid residue in the dystrophin proteins. (C) In the wild type (1344 Leu), the leucine residue forms 5 hydrogen bonds with 4 surrounding amino acid residues (1327 Ala, 1323 Ile, 1340 Ile, and 1348 Asn). (D) After the mutation to phenylalanine, the phenyl ring restricts its interactions, allowing the formation of only 3 hydrogen bonds with 2 surrounding amino acid (1340 Ile, and 1348 Asn) residues. This leads to protein destabilization.

The development of cardiovascular events and HF in PA patients is associated with several risk factors, including metabolic disturbances.^[48–50]^ However, according to current epidemiological data, the prevalence of HF in PA patients is higher than that in patients with essential hypertension (0.6–4.1% vs 1.2%).^[25]^ Yet, it is not universally observed, and the number of reported cases in clinical practice remains limited. In our patient, who is relatively young and lacks metabolic risk factors, whole-exome sequencing was conducted to identify other potential etiologies. A hemizygous mutation in exon 29 of the *DMD* gene was identified in this patient. The *DMD* gene, located on Xp21 and encoding dystrophin, is responsible for a spectrum of dystrophinopathies affecting both skeletal and cardiac muscle. In this patient, the mutation appears to selectively affect the heart without skeletal muscle involvement, as suggested by normal creatine kinase levels and unremarkable electromyogram findings. Previous studies have demonstrated that *DMD*-associated XLDCM may manifest predominantly with cardiac disease,^[51]^ and the phenotype in this case is most consistent with XLDCM. The patient’s mother was identified as a carrier but exhibited no clinical manifestations, with a normal echocardiographic LVEF. It is well recognized that female carriers of X-linked dystrophinopathies may remain clinically asymptomatic due to favorable patterns of X-chromosome inactivation. The clinical heterogeneity of dystrophinopathies is determined by mutation type and tissue-specific expression. Out-of-frame or nonsense mutations typically result in DMD, characterized by near-complete loss of dystrophin, severe early-onset dystrophinopathies. In-frame deletions allow production of partially functional dystrophin, leading to BMD, which is associated with later-onset and milder skeletal muscle and cardiac involvement. By contrast, XLDCM, most often linked to promoter or splicing mutations, selectively impairs cardiac dystrophin isoforms while sparing skeletal muscle, thereby producing isolated, early-onset DCM. Based on previous case reports, *DMD* mutations associated with XLDCM or with mild BMD concomitant with DCM can be classified into 3 categories: variants in the muscle promoter-exon 1 region, which abolish the cardiac muscle isoform of dystrophin,while skeletal isoforms may be preserved through compensatory expression; mutations spanning exons 2 to 8 (encoding the actin-binding domain) and exons 45 to 55 (a mutational hotspot within the rod domain). These mutations destabilize dystrophin, impair actin interaction, and disrupt sarcolemmal integrity, and other variants, including insertional mutations in intron 11, missense mutations in exons 9, 29, and 67, and frameshift mutations in exon 27. Although less common, these variants similarly compromise dystrophin expression or stability and may selectively impair cardiac muscle.^[10,11]^ We then used in silico prediction programs to evaluation its pathogenicity of the mutation. Sorting intolerant from tolerant indicated that the mutation would affect protein function, while PolyPhen-2 classified it as probably damaging. To further assess structural consequences, we employed AlphaFold 2 to predict the 3-dimensional structure of both the normal and mutated dystrophin proteins and visualized them with PyMOL (Fig. 5). Based on these analyses, we hypothesized that the patient’s pathogenesis was caused by structural and comformational changes in the protein. In the wild type (1344 Leu), the leucine residue could form 5 hydrogen bonds with surrounding amino acid residues, stabilizing the protein and enabling normal function. However, substitution with phenylalanine restricted intermolecular interactions, permitting only 3 hydrogen bonds with 2 neighboring residues, thereby destabilizing the protein. The patient’s subtle initial cardiac manifestations suggest that the mutation site may not reside within a critical structural domain of dystrophin, although it might alter protein conformation and stability.

Nevertheless, in the context of PA, chronic hyperaldosteronism may have acted as a pathophysiological modifier, unmasking and potentially accelerating the phenotypic expression of DCM. In PA, autonomous aldosterone hypersecretion persistently activates MR-dependent pathways, promoting sodium retention, hypertension, and direct pro-fibrotic and pro-inflammatory remodeling of the myocardium. Variants in the dystrophin gene (*DMD*) are thought to compromise cardiomyocyte membrane integrity, resulting in intracellular calcium overload, oxidative stress, and sustained inflammation.^[52]^ We therefore propose that, in this case, the interaction between an adverse endocrine milieu and an underlying genetic susceptibility acted synergistically to unmask the cardiac phenotype and hasten the progression of HF.

This report has several limitations. First, the pathogenicity of the identified *DMD* missense variant could not be fully elucidated, as muscle biopsy, functional assays were not performed. Therefore, the variant should be interpreted as a potential genetic modifier of HF severity in the context of PA, rather than a definitive causal factor. Second, as this is a single-patient report, the findings cannot be generalized yet, further research is warranted to clarify the interaction between excessive aldosterone and genetic cardiomyopathies, and to determine whether hyperaldosteronism may act as a “second hit” accelerating cardiac disease in genetically susceptible individuals.

To sum up, we report the case of a 39-year-old patient with PA who presented with early-onset and New York Heart Association Class IV HF. Following surgical intervention, the patient achieved biochemical and clinical remission of PA and significant improvement in cardiac function. This case highlights that early and appropriate treatment of PA complicated by HF can mitigate the effects of hyperaldosteronism, maximal recovery of cardiac function. Furthermore, it underscores the importance of early identification of underlying primary cardiac genetic disorders or other potential causes in PA patients with early-onset HF, rather than attributing HF solely to PA.

## Acknowledgments

We would like to thank Dr Zhaoxu Huang for her assistance in the echocardiographic analysis.

## Author contributions

**Conceptualization:** Xiaoxiao Song, Siwei Qian, Minyue Jia.

**Data curation:** Hanxiao Yu, Minzhi He, Hao Yu, Ka I. Cheok, Liya Lin, Zhichao Dong, Xin Pan.

**Methodology:** Xiaoxiao Song, Chao Zheng, Jiaming Wen, Xiaohong Xu.

**Writing – original draft:** Xiaoxiao Song, Siwei Qian.

**Writing – review & editing:** Xiaoxiao Song, Minyue Jia.

## Supplementary Material




