# Supplementary material for: Primary aldosteronism complicated by early-onset heart failure in a young male with a coexisting DMD variant: A case report and literature review
Source: Medicine (Baltimore). 2025 Nov 7;104(45):e45443. doi: 10.1097/MD.0000000000045443 (PMC12599770; doi:10.1097/MD.0000000000045443)
Supplement: Supplementary file 1 [file medi-104-e45443-s001.docx]

Table S1. Blood and urine test results on admission.

| **Blood Count** | Result | Reference range |
| --- | --- | --- |
| Leukocytes (×10^9^/L) | 7.1 | 4.0-10.0 |
| Hemoglobin (g/L) | 146 | 131-172 |
| Platelets (×10^9^/L) | 168 | 100-300 |
| **Inflammatory indexes** |  |  |
| Hs-CRP (mg/L) | 10.4 ↑ | <5.0 |
| PCT (ng/mL) | 0.16 | <0.5 |
| **Cardiac markers** |  |  |
| LDH (U/L) | 260 ↑ | 120-250 |
| Creatine kinase (U/L) | 102 | <164 |
| Creatine kinase-MB (U/L) | 13 | <24 |
| Pro-BNP (pg/mL) | 7297 ↑ | <125 |
| **Serum chemistry** |  |  |
| FPG (mmol/L) | 4.6 | 3.9-6.1 |
| Albumin (g/L) | 34.7 ↓ | 35.0-52.0 |
| Creatinine (μmol/L) | 187.3 ↑ | 57.0-98.0 |
| eGFR (mL/min) | 38 ↓ | >90.00 |
| Sodium (mmol/L) | 143.6 | 137.0-147.0 |
| Potassium (mmol/L) | 2.6 ↓ | 3.5-5.3 |
| **Blood lipids** |  |  |
| HDL-C (mmol/L) | 0.75 ↓ | 0.90-2.19 |
| LDL-C (mmol/L) | 1.9 | <3.4 |
| TC (mmol/L) | 3.06 | 3.00-5.70 |
| TG (mmol/L) | 0.76 | <1.70 |
| **HbA1c** |  |  |
| HbA1c (%) | 6.2 ↑ | 4.0-6.0 |
| **PTH** |  |  |
| Parathyroid hormone (pg/mL) | 59.15 | 15.00-65.00 |
| **Urinalysis** |  |  |
| Albumin-to-creatinine ratio (mg/g) | 403.6 ↑ | <25.0 |
| Protein | 1+ (0.5g/L) ↑ | - |
| Glucose | - | - |
| 24-hour uric potassium (mmol/24h) | 43.58 | 25.00-100.00 |
