# Supplementary material for: Primary aldosteronism complicated by early-onset heart failure in a young male with a coexisting DMD variant: A case report and literature review
Source: Medicine (Baltimore). 2025 Nov 7;104(45):e45443. doi: 10.1097/MD.0000000000045443 (PMC12599770; doi:10.1097/MD.0000000000045443)

Figure S1. Serial echocardiographic findings before and after adrenalectomy. (A) At presentation: LV dilation (LVIDd 62 mm, LVEDV 186 mL) with markedly reduced function (LVEF 18.3%). (B) 1 month postoperatively. (C) At 4 months: improved systolic function (LVEF 48.5%) with reduced LV size (LVIDd 56.1 mm). (D) At 8 months: reverse remodeling with smaller LV cavity (LVIDd 54.1 mm, LVEDV 95.8 mL) and sustained recovery (LVEF 45.4%).


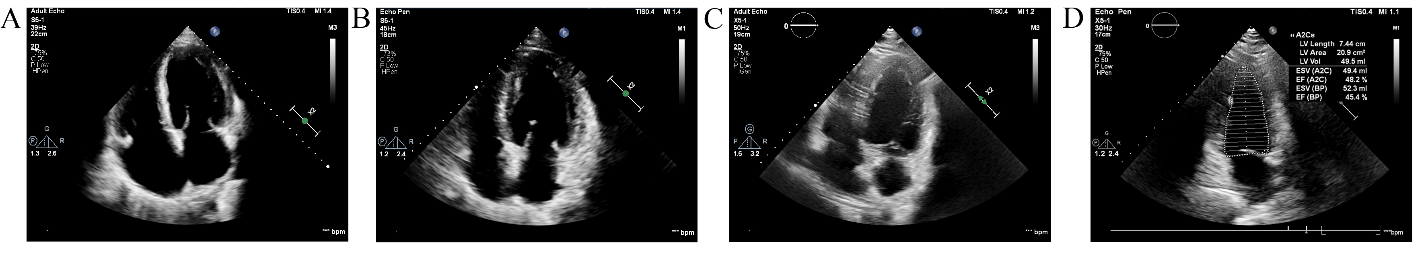

Supplement: Supplementary file 2 [file medi-104-e45443-s002.docx]
